# Identification and functional characterization of a novel arginine/ornithine transporter, a member of a cationic amino acid transporter subfamily in the *Trypanosoma cruzi* genome

**DOI:** 10.1186/s13071-015-0950-y

**Published:** 2015-06-25

**Authors:** Cristina Henriques, Megan P. Miller, Marcos Catanho, Técia Maria Ulisses de Carvalho, Marco Aurélio Krieger, Christian M. Probst, Wanderley de Souza, Wim Degrave, Susan Gaye Amara

**Affiliations:** Fundação Oswaldo Cruz, Fiocruz-Mato Grosso do Sul, Rua Gabriel Abrão 92-Jardim das Nações, Campo Grande, MS 89081-746 Brazil; National Institute of Mental Health, NIH Building 10 Center Driver, Room 4N222, MSC 1381, Bethesda, MD 20892-1381 USA; Department of Neurobiology, University of Pittsburgh School of Medicine, Pittsburgh, PA 15260 USA; Fiocruz, Instituto Oswaldo Cruz, Laboratório de Genômica Funcional e Bioinformática, Av. Brasil 4365, Manguinhos, 21040-900 Rio de Janeiro, RJ Brazil; Instituto de Biofísica Carlos Chagas Filho-UFRJ, CCS-Bloco G-Laboratório de Ultraestrutura Celular Hertha Meyer, Rio de Janeiro, RJ 21949-900 Brazil; Nucleo de Biologia Estrutural e Biomagens, Universidade Federal do Rio de Janeiro-CENABIO, Rio de Janeiro, RJ Brazil; Instituto Nacional de Ciência e Tecnologia em Biologia Estrutural e Biomagens-INBEB, Rio de Janeiro, Brazil; Instituto Carlos Chagas-ICC-FIOCRUZ, Curitiba, PR 81350-010 Brazil

**Keywords:** Arginine, Ornithine, Transporter, Protozoan, Trypanosomatids, *T. cruzi*, Parasite

## Abstract

**Background:**

*Trypanosoma cruzi*, the etiological agent of Chagas disease, is auxotrophic for arginine. It obtains this amino acid from the host through transporters expressed on the plasma membrane and on the membranes of intracellular compartments. A few cationic amino acid transporters have been characterized at the molecular level, such as the novel intracellular arginine/ornithine transporter, TcCAT1.1, a member of the TcCAT subfamily that is composed of four almost identical open reading frames in the *T. cruzi* genome.

**Methods:**

The functional characterization of the TcCAT1.1 isoform was performed in two heterologous expression systems. TcCAT subfamily expression was evaluated by real-time PCR in polysomal RNA fractions, and the cellular localization of TcCAT1.1 fused to EGFP was performed by confocal and immunoelectron microscopy.

**Results:**

In the *S. cerevisiae* expression system, TcCAT1.1 showed high affinity for arginine (K_*m*_ = 0.085 ± 0.04 mM) and low affinity for ornithine (K_*m*_ = 1.7 ± 0.2 mM). *Xenopus laevis* oocytes expressing TcCAT1.1 showed a 7-fold increase in arginine uptake when they were pre-loaded with arginine, indicating that transport is enhanced by substrates on the trans side of the membrane (trans-stimulation). Oocytes that were pre-loaded with [^3^H]-arginine displayed a 16-fold higher efflux of [^3^H]-arginine compared with that of the control. Analysis of polysomal RNA fractions demonstrated that the expression of members of the arginine transporter TcCAT subfamily is upregulated under nutritional stress and that this upregulation precedes metacyclogenesis. To investigate the cellular localization of the transporter, EGFP was fused to TcCAT1.1, and fluorescence microscopy and immunocytochemistry revealed the intracellular labeling of vesicles in the anterior region, in a network of tubules and vesicles.

**Conclusions:**

TcCAT1.1 is a novel arginine/ornithine transporter, an exchanger expressed in intracellular compartments that is physiologically involved in arginine homeostasis throughout the *T. cruzi* life cycle. The properties and estimated kinetic parameters of TcCAT1.1 can be extended to other members of the TcCAT subfamily.

## Background

Protozoans have the capacity to synthesize only a few amino acids; thus, they depend on external sources to supply them with amino acids, the transport of which is mediated by several plasma membrane carriers. *De novo* synthesis of amino acids is restricted to those produced via short pathways or those derived from metabolic intermediates of glycolysis, the citric acid cycle, or the pentose phosphate pathway [1, 2]. Arginine biosynthesis does not occur in the parasite *Trypanosoma cruzi*, which lacks the enzymes argininosuccinate lyase and argininosuccinate synthase, which are responsible for recycling citrulline to arginine [3, 4]. Consequently, arginine is acquired from the host through biochemically characterized high- and low-affinity transport systems on the parasite plasma membrane [5, 6].

*T. cruzi* is an intracellular and highly invasive pathogen transmitted by bloodsucking insects of the subfamily Triatominae. The metacyclic trypomastigote forms released with vector excrement next to the bite wound can infect nearly all tissues [7], and upon entry into cells, they transform into replicative amastigotes. After several cycles of binary division, they then transform into trypomastigotes, which are released into the bloodstream. Bloodstream trypomastigotes within mammalian hosts can be taken up by bloodsucking insects and transformed into epimastigotes, which replicate in the insect midgut and then develop into pathogenic metacyclic trypomastigote forms [8, 9]. Several intermediate stages are completed, but the primary developmental stages in the *T. cruzi* life cycle involve the epimastigote, amastigote, and infective trypomastigote forms [10, 11].

Arginine requirements can vary according to fluctuating biochemical needs specific to each stage of the parasite’s life cycle. In *T. cruzi,* L-arginine is involved in the production of nitrous oxide, high-energy phosphate compounds and protein biosynthesis. However, the protozoan lacks the enzymes: (*i*) arginase, which converts L-arginine to L-ornithine and urea; (*ii*) ornithine decarboxylase; and (*iii*) arginine decarboxylase. Thus, *T. cruzi* is auxothophic for polyamines and there is no evidence of the urea cycle in *T. cruzi* [12–14]; therefore, the amidino group of amino acids can be transferred to amino acceptors to form guanidine derivatives, which can be phosphorylated by kinases, generating high-energy phosphate compounds [15, 16]. In *T. cruzi,* arginine can be phosphorylated by arginine kinase, producing phosphoarginine, a specific phosphagen and high-energy phosphate compound [17, 18] involved in cell energy storage and in pH and nutritional stress response mechanisms [19]. Arginine kinase is not expressed in mammalian tissues, but in this parasite, it is an important enzyme for arginine metabolism that is inhibited by several arginine analogs. However, to date, only canavanine and homoarginine have been shown to significantly inhibit *T. cruzi* epimastigote growth [18, 20].

The fate of L-arginine in this pathogen likely depends on L-arginine availability, the regulation of metabolic enzymes, and the expression of specific transporters. After crossing the protozoan plasma membrane, arginine can enter into intracellular compartments, such as the acidocalcisome, an organelle enriched in cationic amino acids and polyphosphates [21, 22]. Transporters carry out their functions in a variety of membrane compartments, mediating the efflux and influx of molecules and ions and playing roles in osmotic and membrane potential regulation in protozoans [22–24]. The identification of 60 unique sequences encoding putative amino acid transporters [25] in the *T. cruzi* genome is indicative of the complexity of these proteins and their relevance to cell physiology and metabolism. Despite their importance, few amino acid transporters have been characterized at the molecular level in trypanosomatids [26–30].

Here, we report the molecular and functional characterization of a novel arginine/ornithine transporter from *T. cruzi* using heterologous systems. TcCAT1.1 is a member of a subfamily of cationic amino acid transporters, TcCAT, which is composed of four open reading frames (ORFs) in the *T. cruzi* CL Brener genome. The trans-stimulation property of TcCAT1.1 was examined in *Xenopus laevis* oocytes, and the kinetic parameters of transport were analyzed in a *Saccharomyces cerevisiae* null mutant lacking cationic amino acid transporters [31], which is a versatile expression system that allows for efficient drug screening. Quantification of the expression of TcCAT subfamily members was performed throughout the *T. cruzi* life cycle using quantitative PCR (qPCR). Intracellular localization of this transporter in a network of tubules and vesicles at the anterior region of the protozoan suggests that it plays a role in the transport of arginine from intracellular pools of cationic amino acids.

## Methods

### Parasite cultivation

*Trypanosoma cruzi* epimastigotes, wild-type (Dm28c-WT) and genetically modified parasites expressing EGFP (Dm28c-EGFP) or EGFP-TcCAT1.1 (Dm28c-EGFP-TcCAT1.1), were cultivated in liver infusion tryptose (LIT) medium at 28 °C until the logarithmic stage of growth [32] (Camargo, 1964). The non-infective and replicative epimastigotes were transformed into non-dividing and infective metacyclic trypomastigotes. The process referred to as metacyclogenesis was triggered by exposing *T. cruzi* epimastigotes, at the late exponential growth phase and a cell density of 3 × 10^7^ cells/ml, to nutritional stress by incubation in triatomine artificial urine (TAU) medium containing 190 mM NaCl, 8 mM phosphate buffer, pH 6.0, 17 mM KCl, and 2 mM MgCl_2_ for 2 h and further incubation for 5 days in TAU supplemented with amino acids and glucose (TAU3AAG; 0.035 % sodium bicarbonate, 10 mM L-proline, 50 mM sodium glutamate, 2 mM sodium L-aspartate, and 10 mM glucose) [11]. Metacyclic parasites were used to infect LLC-MK2 cells, and trypomastigotes released from these cells were used to infect mice. Amastigotes were prepared as described [33].

### Infection rate

LLC-MK2 cells were placed on 13 mm round glass cover slips in a 24 well microplate and maintained for 18 h in RPMI 1640 medium supplemented with 5 % FBS at 37 °C in a 5 % CO_2_ atmosphere. Then, the cells were washed and exposed to trypomastigotes of Dm28c-WT, Dm28c-EGFP, or Dm28c-EGFP-TcCAT1.1, maintaining a parasite:host cell ratio of 10:1, in 200 μl of RPMI at 37 °C and 5 % CO_2_. After 4 h, infected cultures were washed to remove non-internalized parasites and maintained for 24, 48 or 72 h in RPMI 1640 medium supplemented with 5 % FBS at 37 °C in a 5 % CO_2_ atmosphere. Infected cells were fixed with Bouin’s solution, washed with 70 % ethanol, washed again with water and stained with Giemsa. Subsequently, cover slips were successively dehydrated in the following acetone–xylol mixtures: (1) 100 % acetone; (2) 70 % acetone–30 % xylol; (3) 30 % acetone–70 % xylol; and (4) 100 % xylol. Next, the cover slips were mounted and sealed onto slides with Entelan® (Merck). The rate of infection and number of parasites per infected cell were quantified in at least 500 cells using a light microscope (Leica Microsystems). Two independent experiments were performed in triplicate.

### Animals and infection

Seven-week-old male BALB/c mice were obtained from the Animal Laboratory Breeding Center at Fundação Oswaldo Cruz (CECAL) and housed for 7 days at the Laboratory of Cellular Ultrastructure-UFRJ under the environmental and sanitary conditions established in the guide for the Care and Use of Laboratory Animals (DHEW publication No. [NIH] 80–23). This project was approved by the Biophysics Institution Committee of Ethics in Animal Research (IBCCF106) according to resolution 196/96 of the National Health Council of the Brazilian Ministry of Health. The experimental groups consisted of BALB/c mice intraperitoneally infected with 10^5^ Dm28c-WT, Dm28c-EGFP, or Dm28c-EGFP-TcCAT1.1 trypomastigotes. Parasitemia was determined in 5 μl of blood obtained from tail snips according to the method of Pizzi-Brenner.

### BLAST search and PCR amplification of TcCAT sequences

To clone putative TcCAT transporter genes, basic local alignment search tool **(**BLAST) searches [34, 35] were performed using amino acid transporter protein sequences from *S. cerevisiae*, *Homo sapiens* and other organisms compiled from the National Center for Biotechnology Information (NCBI) in a query against a *T. cruzi* CL Brener predicted protein sequence database [http://www.tigr.org/tdb/e2k1/tca1/]. Candidate amino acid transporters from *T. cruzi* that shared ≥ 20 % identities with the query sequences and showed ≥ 60 % alignment along the query or hit sequences and an e-value of ≤ 10^−5^ were selected using the software BioParser [36]. The putative amino acid transporter sequences were grouped using CLUSTALW (polydot program), generating approximately 11 groups. The TcCAT subfamily was composed of 4 ORFs distributed in three contigs, according to the GeneDB database [http://www.genedb.org].

The corresponding *T. cruzi* TcCAT coding sequences were amplified from genomic DNA by polymerase chain reaction (PCR) with Platinum *Taq* DNA Polymerase High Fidelity (Invitrogen). The forward primer included the EcoRI restriction site (underlined) and Kozak sequence (italics) (5’CGG AAT TCC G*CC ACC* ATG GAC ACC GAG AGT GGC AAT 3’). The reverse primer contained the XhoI restriction site (underlined) and COOH-terminal region of the ORF (5’ CCG CTC GAG CGG TTA CCG AAC CAC ACC ATA CAG GCT 3’). The resulting PCR-amplified fragment was cloned into a pBAD TOPO TA vector (Invitrogen). The following oligonucleotides were designed for automated sequencing: (1) 5’ GGC TTT CAG ATG AGT GGT GTC 3’; (2) 5’ CAA TCG CGC GGT GAC AAG TGC 3’; (3) 5’ CGA GCG CGA GGC GCA TGA CGC 3’; and (4) 5’ TTG CCT TTT CTG TGG AGT TAT; and the reverse oligonucleotide (5) 5’ GAC ACC ACT CAT CTG AAA GCC 3’(Invitrogen). Automated sequencing was performed with an ABI Prism® 310 Genetic Analyzer (Applied Biosystems) at the Department of Neurobiology, University of Pittsburgh.

### Polysomal RNA Purification

*T. cruzi* polysomes, purified from Dm28c-WT epimastigotes and other life cycle forms, were used for qPCR and microarray analysis. Polysomal RNA was extracted from replicating epimastigotes, from parasites incubated for 2 h in TAU medium, which introduced nutritional stress and triggered *in vitro* metacyclogenesis, and from differentiating parasites incubated in TAU3AAG medium for 3 h, 12 h, 24 h, or 5 days to produce metacyclic trypanosomes. Epimastigotes, other life cycle forms, and metacyclic parasites were centrifuged at 2000 g for 20 min at 4 °C and washed three times with NKM buffer, composed of 140 mM NaCl, 5 mM KCl, 1.5 mM MgCl_2_, and 10 mM Hepes, pH 7.4. Next, parasites were lysed with buffer A, composed of 300 mM KCl, 10 mM MgCl_2_, 10 mM Tris–HCl, pH 7.4, 10 % Nonidet P-40 and 2 M sucrose, followed by centrifugation at 16,000 g for 5 min at 4 °C. To obtain the post-mitochondrial fraction, the supernatant was again centrifuged at 16,000 g for 30 min at 4 °C and layered onto 15 to 55 % sucrose density gradients prepared in buffer B, composed of 300 mM KCl, 10 mM MgCl_2_, 10 mM Tris–HCl, pH 7.4, 100 μg/ml cycloheximide, 10 μM E-64, 1 mM phenylmethylsulfonyl fluoride, and 1 mg/ml heparin, and centrifuged at 200,000 g for 2 h. The pellet containing the polysomal fraction was collected, and RNA was extracted by the hot phenol method and with saturated phenol. Samples containing purified RNA were concentrated by precipitation with one volume of 10 % isopropanol and 3 M sodium acetate, purified with an RNAeasy kit (Qiagen) and stored in liquid nitrogen.

### Relative quantification of TcCAT by real-time PCR (qPCR)

Initially, RNA was amplified due to the low yield of RNA obtained from the polysomal fractions of some *T. cruzi* life stages. Amplified RNA was generated from 1 μg of polysomal RNA and oligo (dT) primers coupled to the T7 promoter (US Biochemical Corp.) using a MessageAmp™ aRNA Amplification Kit (Ambion), according to the manufacturer's instructions. Thereafter, cDNA was synthesized from 1 μg of cRNA by incubation with 400 mM of random primers, RT buffer, dNTPs and reverse transcriptase (IMPROM II, Promega), according to the manufacturer’s recommendations, for 2 h at 42 °C. cDNA was purified and concentrated using a Microcon YM-30 filter (Millipore).

Real-time PCR was performed with a 7500 Real-Time PCR System (Applied Biosystems), and the results were normalized by expression of L9 ribosomal protein and histone H2B, which were amplified with the following primers: TcL9F (5` CCTTCACTGCCGTTCGTTGGTTTG 3`); TcL9R (5` ATGCGAGAGTGCCGTGTTGAT 3`); and TcH2BF (5` CGGTGGTGCGCGTCAACAAGAAGC 3`); TcH2BR (5` CCAGGTCCGCCGGCAGCACGAG 3`), respectively. PCR was performed in a 20–25 μL reaction mixture containing 10 ng cDNA and the recommended amount of SYBR Green Master Mix (Applied Biosystems). All reactions contained 4 pmol of specific primers (TcCATf 5'-CATCATTGGATGGGATGTGG-3' and TcCATr 5'-ATAAAGAGCCCGAGCAGCAG-3'). The PCR conditions were as follows: 10 min at 95 °C, followed by 45 cycles at 95 °C for 15 s, 60 °C for 30 s and 72 °C for 1 min. For SYBR Green-based assay, melting curve analysis was performed after amplification to ensure that the correct product had been obtained by determining its specific melting temperature.

The real-time PCR efficiency rate in the investigated range of 5 to 625 ng cDNA (*n* = 3) was calculated as follows, and was found to exhibit high linearity (r > 0.95): for TcH2B, 2.0 (slope −3.313618); for TcL9, 2.05 (slope −3.210819); and for TcCAT transporter, 1.97 (slope −3.405488). Relative gene expression was determined using the 2 ^-∆∆*C*T^ method [37].

### Expression of TcCAT1.1 in *Saccharomyces cerevisiae*

The TcCLB.506153.10 ORF, TcCAT1.1, was excised from a pBAD TOPO TA vector with EcoRI and XhoI restriction digestion and subcloned into the same sites of a galactose inducible yeast expression vector, pYES2 (Invitrogen). *Saccharomyces cerevisiae* strain HSC100-3C (ATCC# 201221) (MATα *can1 gap1 lyp1 ura3∆*) was transformed with plasmid DNA using a Yeastmaker Yeast transformation system 2 (Bioscience Clontech). Transfected yeasts were selected on 1.5 % agar minimal medium plates without uracil and with glucose and amino acid supplementation as required. Selected colonies were cultivated overnight in liquid minimal medium containing 2 % galactose to induce TcCAT1.1 expression or 2 % glucose as a control. Thereafter, the cells were incubated overnight at 30 °C until they reached an OD_600_ of 0.2, centrifuged at 3000 g for 10 min, transferred to new medium and grown to mid-log phase. The cells were centrifuged at 3000 g for 10 min, the excess liquid was drained, and cellular density was adjusted to an OD_600_ of 2 in the appropriate buffer.

Uptake assays were performed by adding 200 μl of cell suspension (OD_600_ = 2) to a 200 μl aliquot of substrate concentrated two-fold. Following incubation for the required period of time, uptake was stopped by addition of 3 ml of cold water and immediate filtration through a nitrocellulose filter with a pore size of 0.45 μm (Millipore). The tube was washed twice with 3 ml of cold water, and the filter apparatus was washed three times. The radioactivity retained in the filter was quantified with a liquid scintillation counter (Wallac 1400).

Substrate saturation curves were obtained by incubation of induced and repressed yeast cells with increasing concentrations of [^3^H]-arginine or [^14^C]-ornithine substrates (Perkin-Elmer Reagents). The incubation time required for uptake was found to be within the linear range. Apparent *K*_*m*_ and *V*_*max*_ values were calculated by non-linear regression analysis and fitted to the Michaelis-Menten equation (SigmaPlot software, SPSS Science). Substrate competition assays were performed with a 100-fold concentration of competitor relative to that of substrate. The optimal pH range for [^3^H]-arginine uptake was achieved in Krebs buffer without glucose, composed of 146 mM NaCl, 5 mM KCl, 2.5 mM CaCl_2_, 1.2 mM MgCl_2_, 5 mM HEPES and 5 mM MES.

### Expression of TcCAT1.1 in *Xenopus laevis* oocytes

The TcCAT1.1 ORF was restriction digested from a pYES2 vector and subcloned into the KpnI and XbaI restriction sites of an oocyte transcription vector, pOTV2-8 [38], to generate a pOTV2-8/TcCAT1.1 plasmid. After linearization with NotI, the new construct was transcribed *in vitro* with T7 RNA polymerase (Life Technologies, Inc.). Stage V-VI *Xenopus laevis* oocytes were injected with 23 nl of cRNA (~10 ng) or water as a control and incubated in ND96 buffer, 96 mM NaCl, 2 mM KCl, 1.8 mM CaCl_2_, 1 mM MgCl_2_ and 5 mM Hepes, pH 7.5, for 3 days at 16 °C. Uptake of radiolabeled compounds was performed in ND96 buffer. Oocytes were dissolved with 10 % SDS and subjected to liquid scintillation counting. For each data point, the pmoles of internalized labeled substrate were calculated and plotted as a function of incubation time.

Trans-stimulation assays were performed using TcCAT1.1-expressing cRNA-injected oocytes and water-injected oocytes preloaded with 1 mM or 10 mM arginine overnight or after 6 h incubation, respectively. Thereafter, the oocytes were washed twice in ND96 buffer at 4 °C and incubated with [^3^H]-arginine at room temperature for uptake assays. For efflux measurements, oocytes preloaded overnight or for 6 h with 1 μM [^3^H]-arginine were washed twice in ND96 buffer at 4 °C and immediately transferred to ND96 buffer (0.5 ml) to allow for the efflux of radiolabeled compounds. After incubation for the required period of time, an aliquot of incubation buffer was examined in a scintillation counter and the total amount of radiolabel in the buffer was divided by the number of oocytes per well.

### Expression of EGFP-TcCAT1.1 fusion protein in *Trypanosoma cruzi*

To fuse EGFP with the TcCAT1.1 transporter at the NH_2_-terminus, TcCAT1.1 was subcloned into an integrative pTREX vector at the BstXI and XhoI restriction sites [39]. Thereafter, a second digestion of the TcCAT1.1-pTREX construct was performed using XbaI and EcoRI to subclone the EGFP cleaved with NheI and EcoRI from a pEGFPC1 vector (Invitrogen), generating pTREX/EGFP-TcCAT1.1. The EGFP-N-terminal TcCAT1.1 fusion construct was sequenced with an ABI 3730 Genetic Analyzer (Applied Biosystems), using the Fiocruz sequencing platform [40]. As a control, EGFP was cleaved from a pEGFP vector with the NheI and XhoI enzymes (Invitrogen) and cloned in the XbaI and NotI restriction sites of pTREX.

*T. cruzi* epimastigotes of the Dm28c or Y strain were suspended at 1 × 10^8^ cells/ml in electroporation buffer (EPB) containing 137 mM NaCl, 5 mM KCl, 0.7 mM Na_2_HPO_4_, 6 mM glucose, and 21 mM HEPES, pH 7.3. The cellular suspension (400 μl) was mixed with 50 μg of plasmid, placed in a 0.2 cm cuvette and subjected to a pulse of 0.45 kV and 500 μF at room temperature using a Gene Pulser apparatus (BioRad Laboratories) [41]. The cells were re-suspended in LIT medium, and G418 (100 μg/ml) was added at 24 h after transfection. The G418 level was increased from 200 to 500 μg/ml to select stable transformants. Then, epimastigote cloning was performed by serial dilutions in a 96 well plate, and clones were evaluated by fluorescence microscopy (Axyoplan, Carl Zeiss).

### Transport Assays of *Trypanosoma cruzi* expressing EGFP-TcCAT1.1 fusion construct

[^3^H]-arginine uptake was assessed in two clones of Dm28c-EGFP-TcCAT1.1, Dm28c-EGFP, and Dm28c-WT. Epimastigotes were centrifuged at 1500 g for 10 min and washed once with Krebs buffer composed of 146 mM NaCl, 2.5 mM CaCl_2_, 1.2 mM MgCl_2_, 5 mM KCl, and 5 mM HEPES, pH 6.8. After centrifugation at 1500 g for 10 min, the excess liquid was drained, and parasite density was adjusted to 10^8^ epimastigotes/ml in Krebs buffer. [^3^H]-arginine (Perkin Elmer) at a specific activity of 50 Ci/mmol was prepared in Krebs buffer and diluted with cold arginine to achieve the specific activity desired for the various experiments.

Uptake assays were initiated by adding 100 μl of parasite suspension (10^7^ epimastigotes) to a 100 μl aliquot of two-fold-concentrated [^3^H]-arginine for a total volume of 200 μM in a 5 ml tube. Following incubation for 15, 30, 60, or 120 min at room temperature and for 30 sec (0.5 min) on ice (binding), uptake was stopped with 3 ml of cold phosphate-buffered saline (PBS), and the suspension was immediately filtered through a nitrocellulose filter with a pore size of 0.45 μm (Millipore). The tube was washed twice, and the filter apparatus was washed once. The radioactivity retained in the nitrocellulose filter was quantified with a liquid scintillation counter (Packard Tricarb) in 2.5 ml of scintillation liquid (Optiphase HiSafe, Perkin Elmer). Three independent assays were performed in triplicate.

### Subcellular localization of TcCAT1.1 in *Trypanosoma cruzi* epimastigotes

Dm28c-EGFP, Dm28c-EGFP-TcCAT1.1 and Dm28c-WT were centrifuged at 1500 g for 15 min at 4 °C, washed twice with PHEM buffer composed of 5 mM MgCl_2_, 70 mM KCl, 10 mM ethyleneglycol-bis-(*β*-aminoethylether)-*N,N,N`,N`-*tetraacetic acid, 20 mM Hepes, and 60 mM Pipes, pH 7.3, fixed with 4 % paraformaldehyde in PHEM buffer for 15 min on ice, allowed to attach to cover slips that were coated with 0.1 % poly-L-lysine (Sigma), permeabilized in 100 % methanol at −20 °C for 5 min, and blocked with 50 mM NH_4_Cl and 1 % BSA in PHEM buffer at room temperature for 30 min. Then, further incubation was performed for 1 h with the following primary antibodies diluted in blocking buffer: (1) anti-rabbit PPase (1:200), a vacuolar-type proton-pumping pyrophosphatase; (2) anti-rabbit *Tc*RAB7 (1:20) [42]; and (3) anti-mouse GFP (1: 200). After three washes with blocking buffer, the slides were incubated with Alexa Fluor 546-conjugated goat anti-rabbit IgG or Alexa Fluor 546-conjugated anti-mouse IgG secondary antibody (1:500) at room temperature for 1 h and washed three times with blocking buffer and once with PBS. Finally, the nuclei and kinetoplasts were stained with DAPI (5 μg/ml) for 10 min. Cover slips were washed four times with PBS, mounted onto slides and observed using a LEICA TCS-SP5 Confocal Laser Scanning Microscope (CLSM, Leica Microsystems). A lambda scan was performed with a 488 nm or 546 nm wavelength, and images were acquired using 5 nm bandwidth increments from 500 to 700 nm.

### Endocytosis in *Trypanosoma cruzi* epimastigotes

Dm28c epimastigotes expressing EGFP-TcCAT1.1 were centrifuged at 1500 g for 10 min and washed with DMEM medium. Thereafter, the parasites were diluted to 10^7^/ml in DMEM medium and starved for 30 min at 28 °C, followed by a 10 min incubation on ice. Endocytosis assays were performed by incubation of 10^7^ epimastigotes with human transferrin conjugated to Alexa Fluor 546 (50 μg/ml) for 1, 5 or 15 min at 28 °C. Binding of human transferrin conjugated to Alexa Fluor 546 to the epimastigote surfaces was performed by incubation on ice for 30 min. To block the endocytic pathway, after starvation, the epimastigotes were treated with 50 mM ammonium chloride for 30 min at 28 °C. Then, human transferrin conjugated to Alexa Fluor 546 was added to the parasite suspension at 50 μg/ml and incubated for 1 or 5 min at 28 °C. Binding and endocytosis were halted with 1 volume of cold 8 % formaldehyde in PHEM buffer, and then the epimastigotes were centrifuged at 1500 g for 10 min and washed twice with PHEM buffer. The parasites were adhered to poly-L-lysine-coated cover slips, which were then incubated with DAPI, washed with PHEM, mounted onto slides and observed using a LEICA TCS-SP5 Confocal Laser Scanning Microscope (CLSM, Leica Microsystems).

### Immunoelectron microscopy

Dm28c-EGFP-TcCAT1.1 and Dm28c-WT epimastigotes were harvested by centrifugation at 1500 g for 10 min and washed with PHEM buffer, pH 7.3. The parasite pellets were fixed in 0.2 % glutaraldehyde, 4 % paraformaldehyde, and 0.5 % picric acid in PHEM buffer for 1 h at room temperature. They were then washed with PHEM buffer and centrifuged at 2000 g for 10 min, incubated with 100 mM glycine in PHEM buffer for 1 h and washed twice with PHEM buffer, pH 7.3. Next, the samples were dehydrated in ethanol at 4 °C, infiltrated with unicryl resin (BB International, Ted Pella) at −20 °C and polymerized under UV light for 120 h. Ultrathin sections were prepared in nickel grids, and they were incubated in blocking buffer composed of 1 % albumin, 0.02 % Tween20, and 0.5 % fish gelatin in PBS for 1 h. Thin sections were subsequently incubated with 1:50 anti-GFP diluted in blocking buffer for 2 h at room temperature, washed and incubated with 15 nm gold-conjugated goat anti-mouse IgG diluted 1:200 in blocking buffer for 1 h at room temperature. Control reactions using these primary and secondary antibodies were performed with ultrathin sections of Dm28c-WT, and the primary antibody was omitted in control reactions with ultrathin sections of Dm28c-EGFP-TcCAT1.1. After extensive washing, the grids were stained with uranyl acetate and lead citrate. Images of the ultrathin sections were obtained with a Zeiss 900 transmission electron microscope.

For electron microscopy, Dm28c-EGFP-TcCAT1.1, and Dm28c-WT epimastigotes were fixed in 2.5 % glutaraldehyde in 0.1 M cacodylate buffer (pH 7.4) for 1 h at room temperature. They were then post-fixed in 1 % osmium tetroxide and 0.8 % potassium ferrocyanide in 0.1 M cacodylate buffer (pH 7.4) for 1 h at room temperature, washed, dehydrated in acetone, and embedded in Epon. The thin sections were stained with uranyl acetate and lead citrate and observed using a Zeiss 900 transmission electron microscope.

## Results

TcCAT is a subfamily of the amino acid/auxin permease (AAAP) family according to the Transporter Classification Database (TCDB), a curated dataset resource composed of transporter sequences from various organisms [43]. The cationic amino acid transporter TcCAT subfamily members from *T. cruzi* contain 4 ORFs distributed in three contigs, according to GeneDB [http://www.genedb.org]. TcCLB.506153.10 (TcCAT1.1) and TcCLB.506153.20 (TcCAT1.2) are located in the same contig and share 100 % coding sequence identity; however, TcCAT1.2 is located at the end of the contig, suggesting that the missing 76 amino acid N-terminal region may have been lost due to a gap in the genome sequencing data, or to misassembly [44]. TcCLB.506053.10 (TcCAT1.3) is 99 % identical to TcCAT1.1, differing by only one amino acid, possessing a Ser^281^ residue instead of Ala^281^. The fourth isoform, TcCLB.511411.30, shares 98 % identity with TcCAT1.1 and was recently identified as the arginine transporter TcAAP3 from *T. cruzi* [45], displaying six amino acid differences in the sequence alignment, including three residues in the amino terminal region and three within the transporter sequence, Ser^281^ to Ala^281^, Phe^327^ to Leu^327^_,_ and Lys^359^ to Arg^359^.

**Fig. 1:**
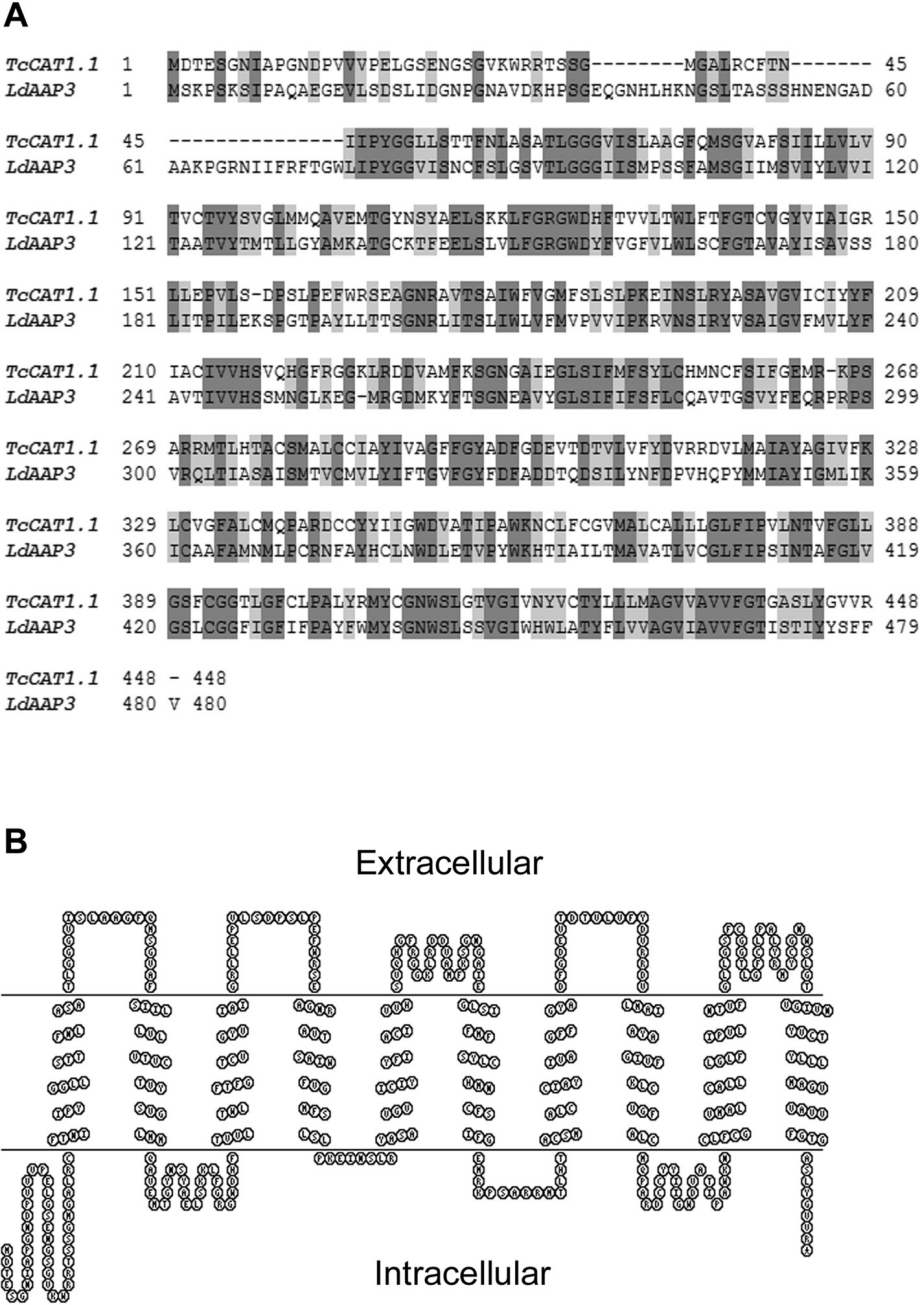
TcCAT1.1 sequencing alignment and membrane topology. **a** Alignment between TcCAT1.1 from *T. cruzi* and LdAAP3 from *L. donovani*. **b** Prediction of TcCAT1.1 membrane topology, as determined using HMMTOP server 2.0

TcCAT1.1 (TcCLB.506153.10 ORF), the isoform that was chosen for functional characterization, possesses 43.6 % identity and 62.5 % similarity at the amino acid level to the arginine transporter LdAAP3 from *Leishmania donovani* (Fig. 1a), and HMMTOP server 2.0 [http://www.enzim.hu/hmmtop] predicted that it contains 10 transmembrane helices (Fig. 1b). Subsequently, the neuronal glutamine transporter from *Rattus norvegicus*, the sodium-coupled neutral amino acid transporter, and intracellular vacuolar amino acid transporters 2 (AVT2) and 6 (AVT6) from *S. cerevisiae* were found to be high-scoring hits in a BLASTP search of the TCDB database (~20 % identity and ~41 % similarity).

The TcCAT subfamily also shares 10 % identity and 23 % similarity at the amino acid level with the human cationic amino acid transporters hCAT-1, hCAT-2A, and hCAT-3. The residues Glu^369^ and Asn^381^ are associated with increased affinity for L-ornithine, L-arginine, and L-lysine in hCAT-1, hCAT-2B, and hCAT-3, and a region of 80 amino acid sequences in length in hCAT members is associated with trans-stimulation properties. The human transporter isoform hCAT-2A, which displays lower affinity for substrates, has an Arg residue in place of Glu^369^, and the Asn^381^ residue is missing [46]. The residues Glu^369^ and Asn^381^, which are responsible for substrate affinity in hCAT members, were not identified in TcCAT1.1, suggesting that other amino acids are responsible for substrate affinity and specificity.

### Functional characterization in *Saccharomyces cerevisiae*

The TcCAT1.1 isoform, one of 4 nearly identical ORFs, was selected for further functional characterization using an *S. cerevisiae* mutant deficient in the following three cationic amino acid transporters: Can1, which transports arginine; Gap1, which transports all amino acids; and Lyp1, the substrates of which are arginine and lysine.

**Fig. 2:**
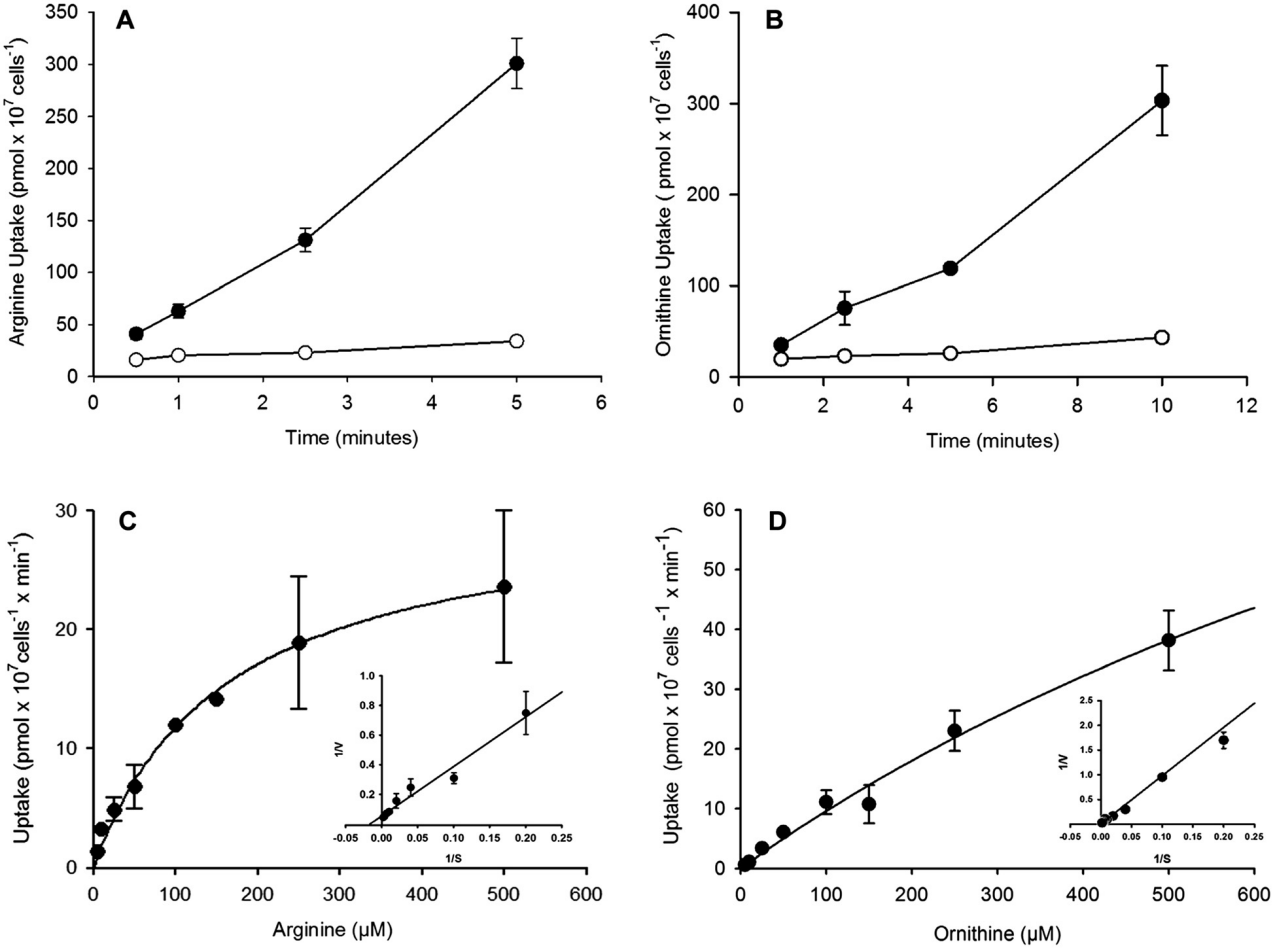
Substrate saturation curves for [^3^H]-arginine and [^14^C]-ornithine in the *Saccharomyces cerevisiae* expression system. **a** and **b** are time course curves in (●) galactose-induced and (○) glucose-repressed yeast cells. **c** and **d** depict the concentration dependence of the [^3^H]-arginine and [^14^C]-ornithine uptake rates in linear ranges. Data from control assays with glucose-repressed cells were subtracted from those from assays with galactose-induced cells, and a Lineweaver-Burk plot of the data is shown in the inset. The results are presented as the mean ± SD of one experiment performed in triplicate

To establish conditions for *K*_*m*_ estimation, time-course curves were generated for each substrate concentration at 0, 0.5, 1, 2.5, 3, 5, and 10 min to assure that the assays were performed within the linear range of substrate uptake (Fig. 2a, b). To investigate relative affinity for arginine, substrate saturation curves were generated using TcCAT1.1-transformed yeast, and an apparent *K*_*m*_ of approximately 0.085 ± 0.04 mM and *V*_*max*_ of 19.7 ± 9.3 pmol x 10^7^ yeast^−1^ x min^−1^ were determined after three trials (Fig. 2c). Ornithine, a lower-affinity substrate for TcCAT1.1, displayed a *K*_*m*_ in the range of 1.7 ± 0.2 mM and a *V*_*max*_ of 83 ± 58.4 pmol × 10^7^ yeast^−1^ x min^−1^, as determined after two trials (Fig. 2d). A comparison of *V*_*max*_/*K*_*m*_ revealed that arginine was a better substrate than ornithine for TcCAT1.1 (Table 1). In the *S. cerevisiae* heterologous system, arginine uptake as mediated by TcCAT1.1 was sensitive to changes in pH. Higher rates of transport were observed at lower pH levels (pH 5 to 6.5), but at pH 8, the transport rate dropped to approximately 50 % of the maximal rate.

**Table 1:** *K*_*m*_ and *V*_*max*_ values for TcCAT1.1 expressed in *Saccharomyces cerevisiae*

Substrate	*V*_*max*_ (pmol.10^7^ yeast^−1^.min^−1^)	*K*_*m*_ (mM)
Arginine	19.7 ± 9.3	0.085 ± 0.04
Ornithine	83 ± 58.4	1.723 ± 0.24

An initial screen for other possible substrates was performed by conducting competition assays to assess [^3^H]-arginine uptake with 100-fold excess of competitor relative to the substrate concentration. Uptake of 10 μM [^3^H]-arginine was used as a reference to estimate the percentage of [^3^H]-arginine uptake in the presence of 1 mM competitors after 30 min of incubation at room temperature (see Table 2). This approach demonstrated that canavanine is another possible substrate because it competes with or inhibits approximately 70 % of arginine uptake. The other tested compounds moderately inhibited arginine uptake (<35 %), implying that they might be lower-affinity substrates. For example, ornithine inhibited arginine uptake by 10 % and displayed an apparent *K*_*m*_ for uptake in the millimolar range (Tables 1 and 2). A variety of amino acids and other inhibitors (methionine, cysteine, cysteic acid, phenylalanine, tyrosine, tryptophan, β-alanine, isoleucine, 4-guanidino butyrate, putrescine, spermidine, N-N dimethyl arginine, 2,3-diamino propionic acid, 2,4-diamino-N-butyric acid, alpha methyl amino isobutyric acid, N-methylaminoisobutyric acid, 2-aminobicyclo heptane-2-carboxylic acid, 2,3-diaminopropionic acid, and pipecolic acid) were tested and showed no competition/inhibition of arginine uptake by TcCAT1.1. Notably, glutamine, asparagine, and histidine, which are substrates for N-system transporters, and the high-scoring hits determined by the BLAST search, were not found to be the main substrates for TcCAT1.1 (Table 2).

**Table 2:** Screening for potential substrates of TcCAT1.1 in *Saccharomyces cerevisiae*

Competitor 1mM	[^3^H]-Arginine uptake(%)
Arginine	18.3 ± 7.9
Canavanine	28.9 ± 6.3
Agmatine	86.1 ± 23.0
Ornithine	88.7 ± 6.4
Citrulline	106.8 ± 22.8
Homoarginine	65.5 ± 12.1
Lysine	78.0 ± 26.7
Histidine	67.3 ± 8.8
Aspartic acid	93.9 ± 5.6
Glutamic acid	93.2 ± 13.5
Asparagine	78.0 ± 6.1
Glutamine	74.2 ± 25.6
Proline	72.3 ± 12.1
Leucine	67.7 ± 16.2
Alanine	67.9 ± 2.6
Spermine	65.0 ± 6.6

To determine whether lysine was a potential substrate, we attempted to directly measure [^3^H]-lysine uptake in *S. cerevisiae* and in *X. laevis* oocytes, but found increased background for [^3^H]-lysine in both systems. We therefore performed competition assays of [^3^H]-arginine uptake with 100-fold excess of cold lysine and found that the uptake of 10 μM [^3^H]-arginine in the presence of 1 mM lysine was approximately 80 % of that without the cold competitor (Table 2), suggesting that lysine could be another lower-affinity substrate for TcCAT1.1, in addition to ornithine.

### Characterization in *Xenopus laevis* oocytes

**Fig. 3:**
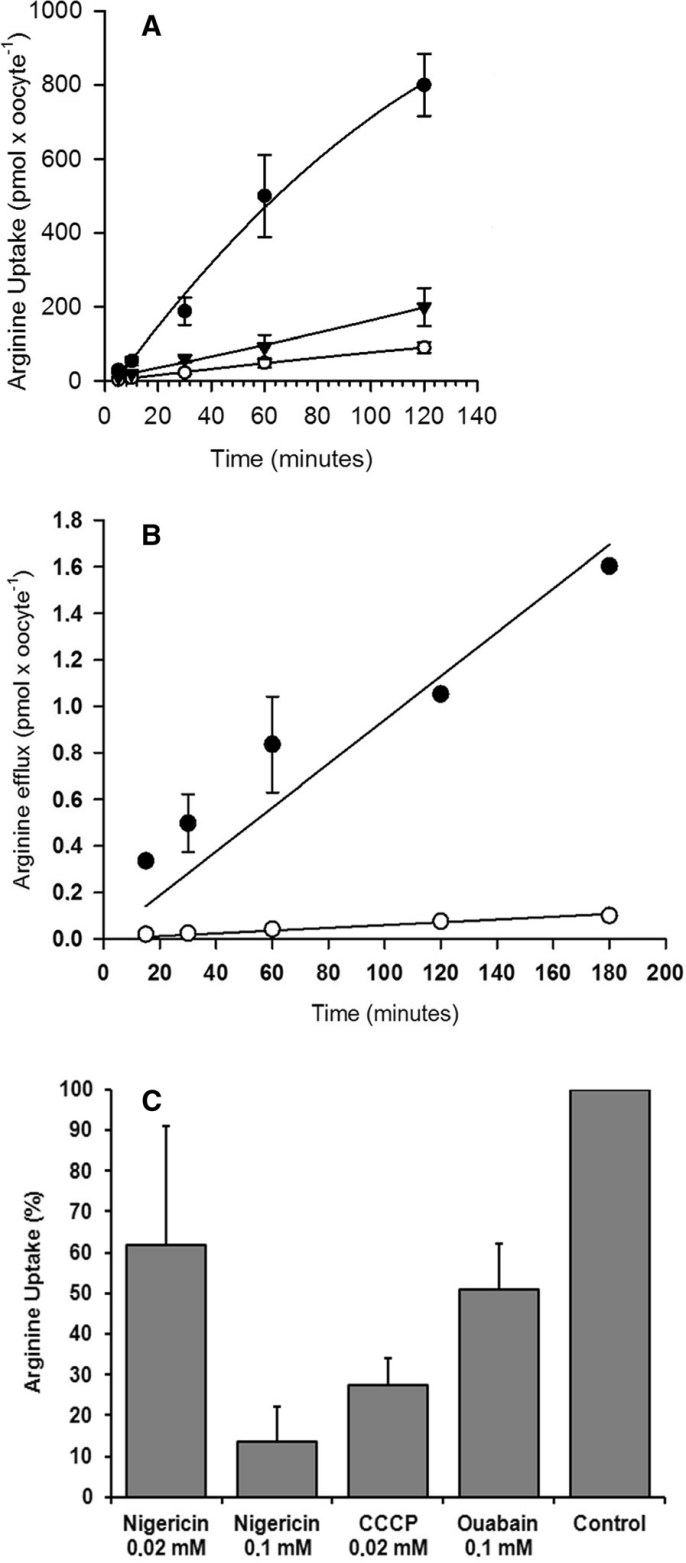
Trans-stimulation in *Xenopus laevis* oocytes expressing TcCAT1.1. Oocytes were pre-loaded overnight with 1 mM arginine. Subsequently, uptake assays were performed by incubating oocytes expressing TcCAT1.1 with 100 μM [^3^H]-arginine. **a** TcCAT1.1-injected oocytes that were pre-loaded with arginine (●); TcCAT1.1-injected oocytes that were not pre-loaded with arginine (▼); and water-injected oocytes, used as controls (○). **b** Efflux of [^3^H]-arginine or arginine sub-products from oocytes injected with TcCAT1.1 cRNA (●) and from control oocytes injected with water (○). Oocytes were pre-loaded for 6 h with 1 μM [^3^H]-arginine in ND96 buffer. **c** Effect of proton uncouplers on [^3^H]-arginine uptake in pre-loaded oocytes expressing TcCAT1.1

Next, a series of experiments were performed using *Xenopus* oocytes to investigate whether TcCAT shares basic properties with other previously characterized cationic amino acid transporters. Trans-stimulation, attributable to the stimulation of [^3^H]-arginine uptake by an intracellular substrate, was observed in oocytes expressing TcCAT1.1 (Fig. 3a). To investigate the magnitude of this phenomenon, oocytes injected with TcCAT1.1 cRNA and those injected with water were pre-loaded to equilibrium by incubation with 1 mM arginine overnight or 10 mM arginine for 6 h in ND96 buffer. The uptake of radiolabeled [^3^H]-arginine in the pre-loaded oocytes was approximately 7-fold higher in the TcCAT1.1-expressing oocytes than in the controls. The oocytes that were not pre-loaded with arginine displayed only a 2- to 3-fold increase in [^3^H]-arginine uptake compared with the water-injected oocytes (Fig. 3a). The trans-stimulation effect was observed in the oocytes pre-loaded with arginine, but not in those pre-loaded with lysine, leucine, or alanine at 10 mM for 6 h. In the latter cases, [^3^H]-arginine uptake was stimulated by only 2- to 3-fold in oocytes expressing TcCAT1.1 compared with that in the controls (data not shown). The effect observed in the oocytes pre-loaded with arginine suggests that the increased uptake of [^3^H]-arginine is driven by arginine present on the trans side of the membrane (Fig. 3a). The transport rate was consistently higher in the oocytes expressing TcCAT1.1 pre-loaded with arginine, reaching 3.65 ± 2.65 pmol x min^−1^, compared with that in the oocytes that were not pre-loaded, in which it reached 0.60 ± 0.51 pmol x min^−1^ (*n* = 6). Arginine uptake driven by trans-stimulation was observed with [^3^H]-arginine at 100, 250, and 500 μM substrate concentrations (data not shown).

Next, the efflux of [^3^H]-arginine was investigated in TcCAT1.1-expressing oocytes pre-loaded with 1 μM [^3^H]-arginine. The time course of [^3^H]-arginine efflux in oocytes that were pre-loaded for 6 h revealed a rate of 10 ± 2 fmol x min^−1^ in TcCAT1.1-expressing oocytes and a rate of 0.6 ± 0.08 fmol x min^−1^ in water-injected oocytes. In oocytes pre-loaded overnight with [^3^H]-arginine, the efflux rate was approximately 19 ± 3 fmol x min^−1^ in TcCAT1.1-expressing oocytes and 1 ± 0.2 fmol x min^−1^ in controls. This rate remained linear for 3 h and was at least 16-fold higher in the TcCAT1.1-expressing oocytes compared with that in the controls, which were water-injected oocytes pre-incubated with [^3^H]-arginine (Fig. 3b).

To evaluate the effect of ionophores on [^3^H]-arginine uptake as mediated by TcCAT1.1, oocytes pre-loaded overnight with 1 mM arginine were pre-incubated for 20 min with 20 μM carbonyl cyanide m-chlorophenylhydrazone (CCCP; a proton ionophore), with 20 μM or 100 μM nigericin (a proton-potassium antiporter), or with 100 μM ouabain (a sodium-potassium ATPase inhibitor) in ND96 buffer. Uptake assays were performed for 30 min at room temperature with 100 μM [^3^H]-arginine in ND96 buffer in the presence of each of the three compounds (Fig. 3c). The uptake of [^3^H]-arginine was reduced by nigericin in a dose-dependent manner, resulting in 34.3 ± 18.3 pmol x oocyte^−1^ (*n* = 3) for 20 μM nigericin and 11.1 ± 7.2 pmol x oocyte^−1^ (*n* = 3) for 100 μM nigericin compared with that of the controls, which was 65.0 ± 6.4 pmol x oocytes^−1^ (*n* = 5). In the presence of 20 μM CCCP, the level of [^3^H]-arginine uptake was 17.1 ± 4.1 pmol x oocyte^−1^ (*n* = 4), and with 100 μM ouabain, it was 26.7 ± 11.4 pmol x oocyte^−1^ (*n* = 4). The water-injected oocytes were subjected to the same treatments, and the background was subtracted.

### TcCAT subfamily expression during *T. cruzi* life cycle

The genes encoding members of the *T. cruzi* TcCAT subfamily were found to be differentially expressed by microarray analysis and qPCR. To evaluate gene expression during the *T. cruzi* life cycle and during metacyclogenesis, microarray data from three different metacyclogenesis experiments were analyzed (biological replicas) using polysomal RNA preparations that were hybridized at least twice to the *T. cruzi* microarray (technical replicates). We examined the gene expression levels of TcCAT subfamily members using three different probes, which all showed overexpression of the epimastigote TcCAT genes under nutritional stress and down regulated expression during cellular differentiation. A comparison of the gene expression levels of the TcCAT subfamily members between epimastigotes and amastigotes by microarray revealed no significant differences (Christian M. Probst, personal communication).

**Fig. 4:**
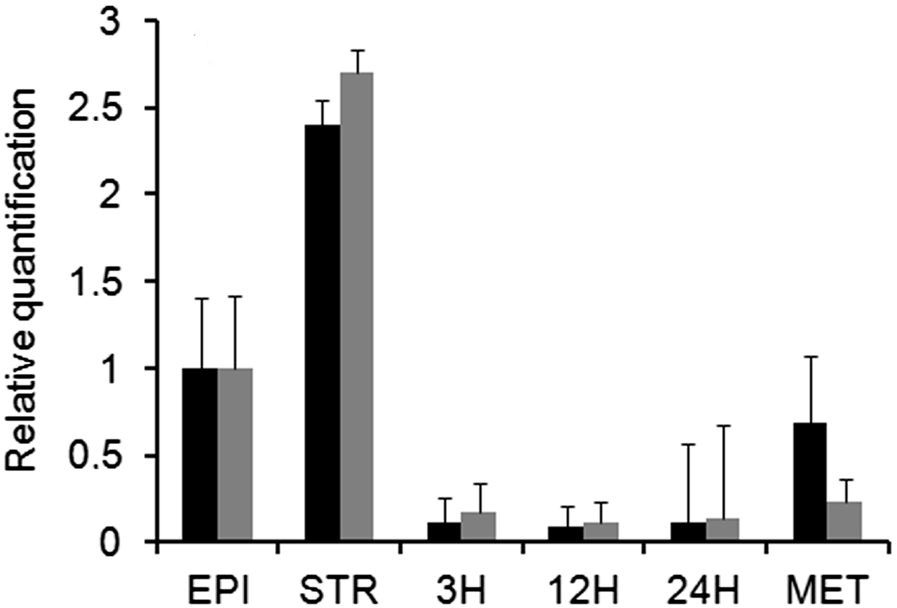
Differential expression of TcCAT subfamily members as determined by real-time polymerase chain reaction. Quantification of polysome-associated mRNA expression during metacyclogenesis >relative to that in epimastigotes in samples obtained from metacyclogenesis experiment. Ribosomal L9(gray) and histone H2B(black) were used as controls to normalize the expression of polysomal mRNAs encoding TcCAT. EPI, replicating epimastigotes; STR, nutritional stress; 3H, parasites after differentiation for 3 h; 12H, parasites after differentiation for 12 h; 24H, parasites after differentiation for 24 h; and MET, metacyclics

To validate the data obtained by microarray and to quantify TcCAT subfamily expression, we performed qPCR using polysomal RNA prepared [47] from epimastigotes that had been exposed to nutritional stress for 2 h in TAU medium, to trigger *in vitro* metacyclogenesis, from parasites undergoing differentiation in TAU3AAG medium for 3, 12, (12H) or 24 h, and from metacyclic trypomastigotes. The values obtained were normalized to those of the mRNAs encoding ribosomal L9 and histone H2B. These results corroborated the previous microarray results, demonstrating that when cells are exposed to nutritional stress in TAU medium, which mimics the urine of the triatomine host, these transporters are upregulated, and the mRNA levels increase after 2 h of starvation (Fig. 4). The expression of the TcCAT subfamily members in epimastigotes under nutritional stress varied during metacyclogenesis; however, both experiments demonstrated increases in the levels of mRNAs associated with polyribosomes. Protein expression is post-transcriptionally regulated in trypanosomes; thus, we evaluated the levels of mRNAs associated with polyribosome clusters, which are sites of protein synthesis, and found increased expression of TcCAT subfamily members.

### *In vitro* and *in vivo* infection rates

To address the physiological contribution of TcCAT1.1 during the protozoan life cycle, an EGFP-TcCAT1.1 N-terminal fusion construct was cloned into a pTREX vector and overexpressed in the Dm28c strain. Transgenic Dm28c-EGFP-TcCAT1.1 epimastigotes were able to differentiate into metacyclic trypomastigotes and infect LLC-MK2 cells and mice. Evaluation of *in vitro* infection of LLC-MK2 cells by optical microscopy of stained cells demonstrated that transgenic parasites have similar rates of infection to those of wild-type parasites, as evidenced by the following findings: (1) after 24 h, Dm28c-EGFP-TcCAT1.1 epimastigotes infected 16 to 25 % of the cells, and Dm28c-WT epimastigotes infected 16 to 27 % of the cells; and (2) after 48 h, these rates dropped to 11 to 24 % and 10 to 18 %, respectively. Indeed, once these cells differentiated into amastigotes, they resumed normal proliferation comparable to that of wild-type parasites, and 1.2 to 1.4 amastigotes per infected cell were detected for both strains (*n* = 2).

As a first step of characterizing infection in mice using transgenic Dm28c-EGFP-TcCAT1.1 parasites, we compared blood parasitemia in mice infected with transgenic Dm28c-EGFP-TcCAT1.1 and Dm28c-WT trypomastigotes, and transgenic Dm28c-EGFP was used as a control. Mice infected with the wild-type strain and with Dm28c-EGFP showed a typical parasitemia curve, which increased up to 20 dpi to 3.7 ± 2.0 × 10^5^ and 5.0 ± 2.2 × 10^5^ trypomastigotes/ml, respectively (*n* = 6). However, in the mice infected with transgenic Dm28c-EGFP-TcCAT1.1 trypomastigotes, parasitemia was lower at 20 dpi, with 6.0 ± 3.9 × 10^4^ trypomastigotes/ml (*n* = 5) but with patent levels of circulating parasites at all time points (not shown), indicative of active infection. Thus, the course of infection may vary between transgenic parasites expressing only EGFP and those expressing EGFP-TcCAT1.1.

### TcCAT1.1 localization in *T. cruzi* and arginine uptake in *T. cruzi* expressing EGFP-TcCAT1.1

**Fig. 5:**
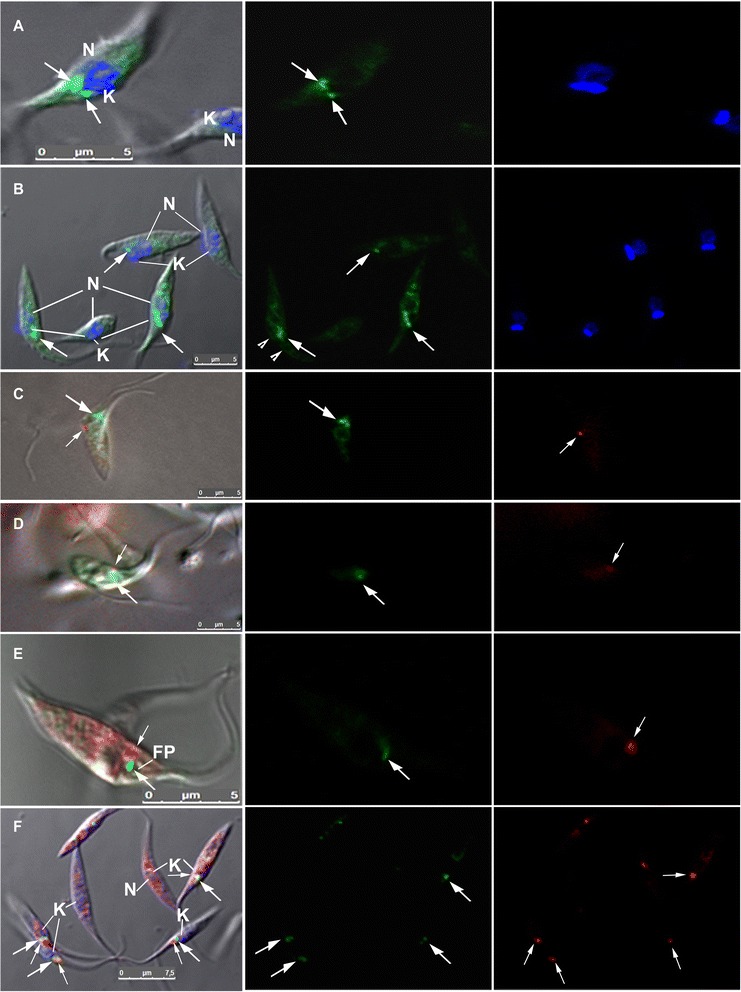
Localization of EGFP-TcCAT1.1 in *Trypanosoma cruzi*. Stable transfectants of Dm28c over-expressing EGFP-TcCAT1.1 were cloned and maintained in 500 μg/ml of G418. Expression was evaluated by confocal microscopy. Panel (**a**) shows the intracellular distribution of EGFP-TcCAT1.1 at the anterior regions of the parasite (arrows). Panel (**b**) shows additional sites of reduced expression following incubation with anti-GFP, including the epimastigote surface (arrow head). Panels (**c**) and (**d**) show endocytosis with transferrin-Alexa Fluor 546 (small arrow) after 1 min in ammonium chloride-treated epimastigotes (**c**) and after 15 min (**d**). Panels (**e**) and (**f**) show the results of labeling with anti-RAB7 (small arrow) to evaluate co-localization of EGFP-TcCAT1.1 (arrow) with the Golgi complex. The left column shows differential interference contrast microscopy, the central column depicts EGFP-TcCAT1.1 expression (green), and the right column shows DAPI labeling of the nucleus and kinetoplast (blue), anti-RAB7, and transferrin-Alexa Fluor 546 tracer (red). N, nucleus; K, kinetoplast; FP, flagellar pocket

The EGFP-TcCAT1.1 fusion construct was expressed in Dm28c because of its high efficiency of *in vitro* metacyclogenesis. Expression of the Dm28c-EGFP-TcCAT1.1 chimera was apparent in epimastigotes (Fig. 5a) and intracellular amastigotes (not shown) but was faint in trypomastigotes (not shown), as visualized by fluorescence microscopy, consistent with TcCAT subfamily expression throughout the *T. cruzi* life cycle, as evaluated by qPCR (Fig. 4). TcCAT1.1 was not expressed in acidocalcisomes, as observed by fluorescence microscopy, and it did not co-localize with the vacuolar-type proton-pumping pyrophosphatase (PPase; not shown); however, we cannot discount the fact that other isoforms that are TcCAT subfamily members are expressed in acidocalcisomes. As illustrated in Fig. 5, EGFP-TcCAT1.1 labeling was predominant in the anterior regions of epimastigotes, and its position relative to the DAPI-stained nucleus and kinetoplast indicated that TcCAT1.1 was located near the flagellar pocket, Golgi complex, and spongiome region, which is a network of vesicles and tubules (Fig. 5a) [48]. Incubation with anti-EGFP reinforced this localization but revealed its distribution in other intracellular compartments, including the endoplasmic reticulum (Fig. 5b).

**Fig. 6:**
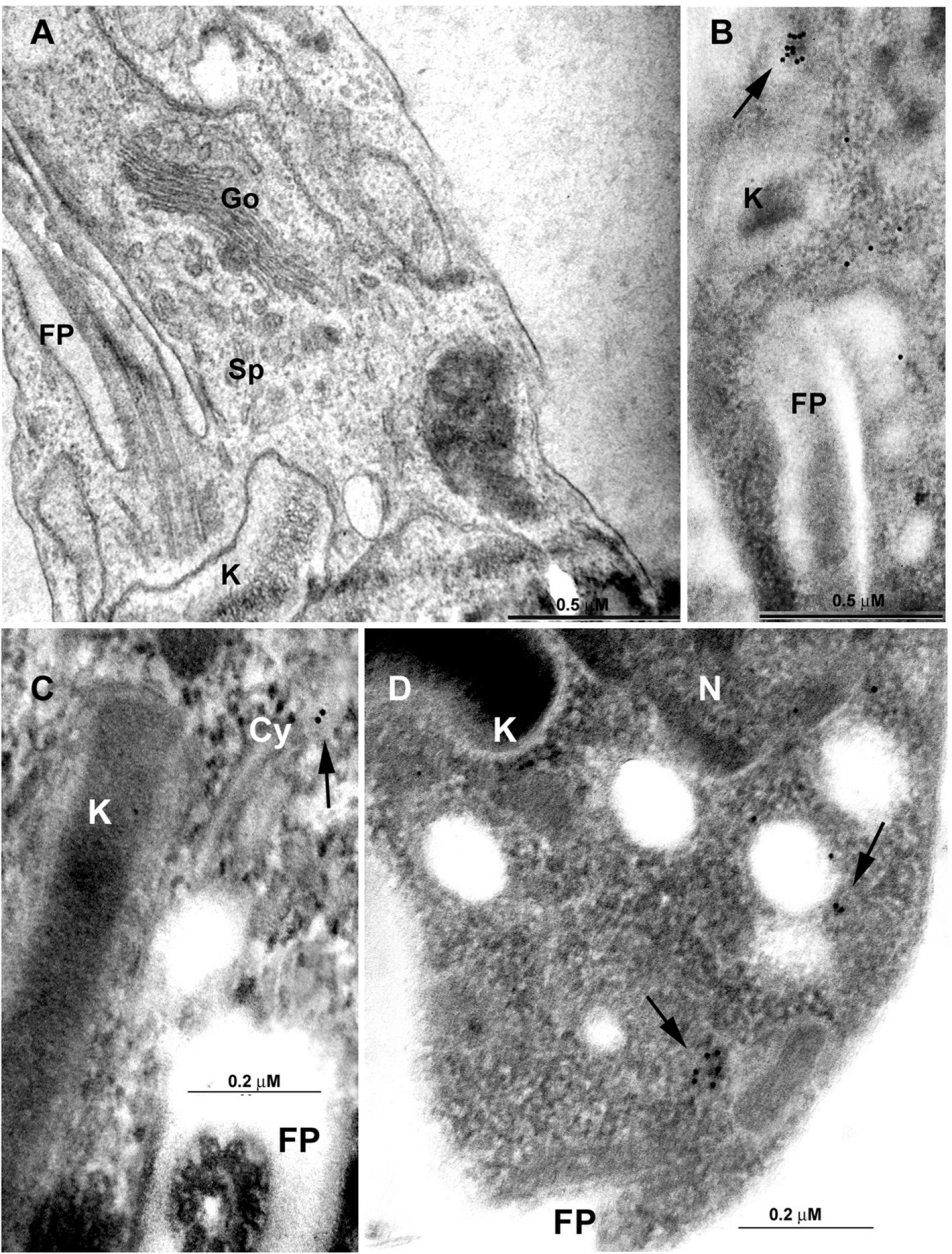
Ultrastructural localization of EGFP-TcCAT1.1. Stable transfectants of Dm28c epimastigotes over-expressing EGFP-TcCAT1.1 were evaluated by transmission electron microscopy. **a** Ultrastructural organization of the epimastigote anterior region embedded in epoxy resin. **b-d** Immunoelectron microscopy to evaluate EGFP-TcCAT1.1 localization in the spongiome region, as observed by gold particle distribution (arrow), after incubation with anti-GFP. N, nucleus; K, kinetoplast; FP, flagellar pocket; Go, Golgi complex; Sp, spongiome; Cy, cytostome

**Fig. 7:**
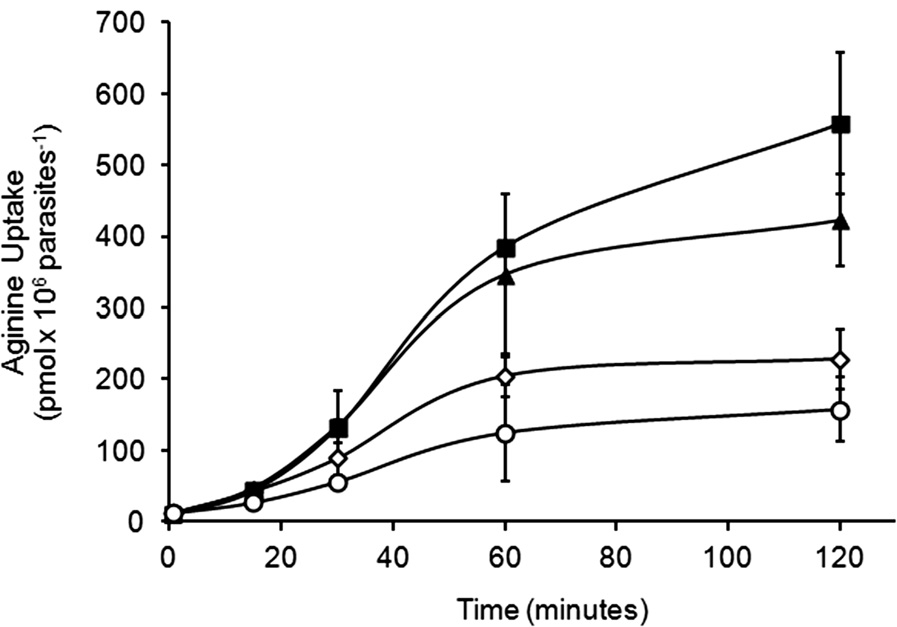
Time course curve of [^3^H]-arginine in *Trypanosoma cruzi* epimastigotes. [^3^H]-arginine uptake was evaluated in two clones of transgenic Dm28c EGFP-TcCAT1.1 epimastigotes (■,▼), in WT Dm28c (◊), and in transgenic Dm28c-EGFP, used as a control (○)

The site of EGFP-TcCAT1.1 expression in the anterior epimastigote region is also where endo- and exocytosis occur; thus, we performed transferrin-Alexa Fluor 546 uptake experiments to verify its co-localization with the endocytic pathway. After incubation for 1 to 15 min with transferrin-Alexa Fluor 546, conjugated transferrin was found in the cytostome, cytophraynx, and early endosome, as previously described [49]. However, it did not co-localize with EGFP-TcCAT1.1 (Fig. 5c, d). In epimastigotes expressing EGFP-TcCAT1.1, the intracellular labeling near the Golgi complex in the anterior region was duplicated during cell division (Fig. 5b), as observed for the GTPase TcRAB7 protein expressed in the Golgi of *T. cruzi* [42]. TcRAB7 labeling was punctuate and was in close vicinity to that of EGFP-TcCAT1.1, juxtaposed to the Golgi complex in vesicles; however, their co-localization was not conclusive (Figs. 5e-f and 6a). EGFP-TcCAT1.1 expression was not observed in the flagellar pocket, cytostome, or Golgi complex, as demonstrated by confocal microscopy (Fig. 5c-f) and immunoelectron microscopy (Fig. 6b-d), and it was mainly found in vesicles at the anterior region, comprising vesicles or lamellae of the spongiome or multivesicular bodies [48]. Importantly, TcCAT1.1 is also expressed in other compartments and in regions of the plasma membrane (Fig. 5b), which could explain the 2- to 3-fold higher uptake of 100 μM [^3^H]-arginine in two clones of Dm28c epimastigotes over-expressing EGFP-TcCAT1 compared with that of the controls, which included EGFP-expressing Dm28c and wild-type Dm28c (Fig. 7).

## Discussion

### Functional characterization in heterologous systems

In *T. cruzi,* most studies of transport systems have focused on global measurements of cellular uptake, and few have characterized the molecular mechanisms underlying these activities by performing molecular cloning and functional characterization in heterologous systems. Herein, we describe the molecular characterization of a novel intracellular arginine/ornithine transporter from *T. cruzi*, TcCAT1.1, a member of the cationic amino acid transporter subfamily TcCAT, which displays high affinity for arginine (*K*_m_ on the order of 0.085 mM) and lower affinities for other cationic amino acids, such as ornithine (*K*_m_ of 1.7 mM) (Fig. 2). The intracellular concentration of L-arginine in human tissues is in the millimolar range (0.1-1 mM) and depends on the cell type, and the plasma levels range from 0.040 to 0.115 mM [50], which is within the range of *K*_m_ values estimated for arginine uptake by TcCAT1.1.

The most relevant arginine transport system in mammals is the Na^+^-independent high-affinity y + system, which includes four well-characterized cationic amino acid transporters, CAT-1, CAT-2A, CAT-2B, and CAT-3. The *K*_m_ values are estimated to be between 70 and 250 μM for L-arginine, L-ornithine, and L-lysine for CAT-1, 40 and 380 μM for CAT-2B, and 2 to 5 mM for CAT-2A when they are expressed in *Xenopus laevis* oocytes [50–55]. We have previously shown that the apparent *K*_*m*_ can vary depending on the system used for functional studies [56] but that the apparent affinity of TcCAT1.1 for arginine closely resembles its affinities for CAT-1 and CAT-2B.

TcCAT1.1 displays several distinct properties compared with the hCAT cationic amino acid transporter family (hCAT-1, hCAT-2A, hCAT-2B, and hCAT-3), and it can thus be explored as a strategy for chemotherapy. Unlike hCATs, which bind with high affinity to several basic amino acids, TcCAT1.1 binds with high affinity to L-arginine. Ornithine and possibly lysine are low-affinity substrates for TcCAT1.1 in *T. cruzi*, as inferred from competition studies performed in *S. cerevisiae* expressing TcCAT1.1 (Table 2). Canavanine is another possible substrate for TcCAT1.1, and it competes effectively with arginine, resulting in 70 % inhibition of arginine uptake (Table 1). Canavanine is an example of a plant-derived product because it is an arginine analog found primarily in the seeds of certain leguminous plants. It displays cytostatic and anti-proliferative effects in *T. cruzi* epimastigote cultures, incorporating into proteins, causing them to be non-functional and structurally aberrant [57]. Moreover, unlike CAT-2B, which shows only 50 % activity at low pH [52], TcCAT1.1 is highly active at a broad range of acidic to neutral pH levels (data not shown), and this broad range of activity can be physiologically important for a transporter that operates as an exchanger and is located in intracellular compartments. Similar findings have been observed for arginine transport mediated by the LdAAP3 transporter from *Leishmania donovani,* which displays optimum activity at pH 5.0, the acidic pH level of the parasitophorous vacuole compartment [27]. Indeed, in oocytes, arginine uptake as mediated by TcCAT1.1 was affected by nigericin, a Na^+^/H^+^ exchanger, and was reduced by 70 % by the ionophore CCCP (Fig. 3c). These findings are similar to those obtained with the yeast vacuolar transporters AVT1-7 [58] and plant/auxin amino acid permeases (AAAP family) from *Arabidopsis thaliana*, which actively transport amino acids into plant cells by an amino acid/H^+^ symport mechanism [59].

TcCAT1.1 is also involved in the trans-stimulation of arginine uptake, and substrate accumulation is activated by the presence of intracellular substrate (Fig. 3a). This characteristic property has been reported previously for members of the human CAT family and for the heterodimeric amino acid transporter hF2hc/y^+^LAT2 [52, 60, 61]. *Xenopus* oocytes actively synthesize proteins; thus, some of the accumulated intracellular arginine can be incorporated into proteins, complicating estimation of the precise concentration of intracellular arginine available for exchange. Nevertheless, the influx rate was consistently more robust in TcCAT1.1-expressing oocytes that were pre-loaded with arginine (Fig. 3a). Arginine influx was in the pmol x min^−1^ range, and equilibrium was achieved after 3 h, whereas the outward flux capacity for arginine was 1000-fold lower (Fig. 3b). Investigating the parameters of arginine efflux in greater detail, including the binding and translocation of substrates from the intracellular side of the membrane, will require more readily manipulatable systems, such as plasma membrane vesicles isolated from *S. cerevisiae*, *T. cruzi* over-expressing the TcCAT1.1 transporter, and liposomes.

### Evaluation of TcCAT1.1 expression in *Trypanosoma cruzi*

TcCAT subfamily mRNA was consistently more abundant in epimastigotes under nutritional stress compared with that in those cultivated in regular medium and in metacyclic trypomastigotes (Fig. 4), suggesting that stage-specific expression is the result of differential mobilization of mRNAs to the polysome fraction, as has been described for other *T. cruzi* genes [62]. Upregulation of TcCAT subfamily members in epimastigotes subjected to starvation can be important for the maintenance of arginine homeostasis during metacyclogenesis and the translocation of arginine from intracellular pools, such as the acidocalcisomes, reservosomes, and other intracellular compartments. Similarly, expression of the LdAAP3 transporter and its transcripts in *L. donovani* has been shown to increase as a function of the durations of starvation and arginine deprivation, ranging from 2 to 4 h, and it is correlated with decreases in the intracellular levels of most amino acids, including arginine [63]. Further, starvation of mammalian cells for amino acids has been shown to enhance CAT-1 activity by increasing CAT-1 mRNA stability and translation [64, 65], thereby increasing CAT-1 abundance.

The differences in arginine metabolism between the human host and *T. cruzi* are associated with the fact that the protozoa have no capacity for arginine biosynthesis, causing it to be entirely dependent on transporters to acquire this amino acid from the environment. As a consequence, it is more vulnerable than host cells to drugs that block or interfere with arginine metabolism or disrupt its transport. Importantly, there are some particularities of arginine metabolism in *T. cruzi* compared with its mammalian host and *Leishmania* species. In *T. cruzi*, arginine is converted to phosphorylated arginine in a reaction catalyzed by arginine kinase, an enzyme that is not known to exist in *Leishmania*. However, in contrast with the biosynthetic pathways of *Leishmania*, polyamine biosynthesis in *T. cruzi* remains controversial because this parasite lacks the enzyme ornithine decarboxylase, and there is conflicting evidence about whether polyamine biosynthesis occurs via arginine decarboxylase [66–68].

Some intracellular compartments serve as storage sites, and arginine and lysine constitute approximately 90 % of the total amino acids in *T. cruzi* acidocalcisomes [22]. However, other compartments of arginine metabolism also contain arginine pools, such as the reservosome and possibly other vesicles and tubules that have not yet been identified, and they represent sites of storage and maintenance of intracellular arginine pools. During metacyclogenesis, TcCAT subfamily expression is upregulated; however, identification of the intracellular localizations of the isoforms TcCAT1.1, TcCAT1.2, and TcCAT1.3 is crucial for the understanding of the dynamics of arginine mobilization under stress conditions and to reveal the locations of other intracellular pools of arginine. To determine the cellular localization of the TcCAT1.1 isoform, EGFP was fused to the N-terminal region of the transporter. Overexpression of this fusion protein in epimastigotes resulted in intracellular labeling observed mainly at the anterior regions of epimastigotes (Fig. 5). A comparison of the pattern of EGFP fluorescence with that of DAPI labeling indicated that TcCAT1.1 was located adjacent to the nucleus and very close to the flagellar pocket. The Golgi complex and the endocytic pathway were excluded as sites of TcCAT1.1 expression (Fig. 5c-f). Because few antibodies can be used to identify discrete compartments, we used electron microscopy and immunocytochemistry to determine the precise localization. The flagellar pocket and Golgi complex were excluded as the expression sites of this transporter; however, a network of tubules and vesicles called the spongiome was observed by immunocytochemistry and is a candidate location of TcCAT1.1 isoform expression (Fig. 6). The spongiome and its surrounding area have been recently described, and its ultrastructure has been elucidated by high-pressure freezing and electron tomography [48]. This region is associated with the intense trafficking of vesicles and exocytosis, and it may play a role in the secretome [69]. Other transporters have been reported to be localized to the anterior region of this parasite, for example, two polyamine transporters have been found in the network of vesicles and tubules surrounding the contractile vacuole [30], and the TcABC1 exchanger has also been identified in this region [26]. Similar to the TcABC1 transporter, which is distributed in intracellular compartments and in regions of the *T. cruzi* plasma membrane [26], TcCAT1.1 is also expressed in different regions, including intracellular compartments and at lower levels in the plasma membrane. This membrane localization could explain the [^3^H]-arginine uptake that was observed following expression of EGFP-TcCAT1.1 by whole epimastigotes (Fig. 7).

## Conclusions

Substrate affinity, trans-stimulation, and the kinetic parameters of arginine/ornithine transport estimated for TcCAT1.1 can be extended to other TcCAT subfamily members, such as TcCAT1.2, TcCAT1.3, and TcAAP3. Previous characterization of the TcAAP3 transporter has revealed that it is also an arginine transporter that is expressed in the anterior region of epimastigotes. Intracellular expression is an important characteristic of members of the TcCAT subfamily of cationic amino acid transporters, and it could account for their translocation of intracellular pools of cationic amino acids. Members of this transporter subfamily characteristically function as exchangers, consistent with the intracellular localization of the TcCAT1.1 isoform, and its up-regulation under nutritional stress suggests that TcCAT transporters are candidates for regulation of cellular homeostasis during metacyclogenesis and during the *T. cruzi* life cycle. The complexity of the expression of the transporters required to obtain arginine and cationic amino acids from the environment is associated with their importance to *T. cruzi* biology. We have performed a BLAST search, identifying 26 putative amino acid transporters. In addition, we have identified another arginine permease in the *T. cruzi* genome using a genome database of annotated sequences [http://www.genedb.org/]; thus, it is reasonable to propose that there might be additional cationic amino acid transporters in *T. cruzi*. An increase in the number of transporters cloned and characterized from *T. cruzi* will provide new insights into the contributions of various transporters to the metabolism of cationic amino acids throughout the life cycle of this parasite.

## Abbreviations

AAAP: Acid/auxin permease
BCH: 2-Aminobicyclo heptane-2-carboxylic acid
BLAST: Basic Local Alignment Search Tool
CCCP: Carbonyl cyanide m-chlorophenylhydrazone
EGFP: Enhanced green fluorescent protein
EPB: Electroporation buffer
LIT: Liver infusion tryptose
NCBI: National Center for Biotechnology Information
NMAIB: N-methylaminoisobutyric acid
NO: Nitric oxide
ORF: Open reading frame
PHEM buffer: 5 mM MgCl_2_, 70 mM KCl, 10 mM ethyleneglycol-bis-(*β*-aminoethylether)-*N,N,N`,N`*-tetraacetic acid, 20 mM Hepes, and 60 mM Pipes, pH 7.3
PPase: Vacuolar-type proton-pumping pyrophosphatase
TAU: Triatomine artificial urine
TAU3AAG: Supplemented with amino acids and glucose
TCDB: Transport Classification Database
